# Spinal and Nervous Diseases, Rheumatism, and Paralysis; or Cases and Observations Illustrating an Improved Treatment

**Published:** 1842-07

**Authors:** 


Art. VIII.—*Spinal and Nervous Diseases, Rheumatism, and Paralysis; or Cases and Observations illustrating an Improved Treatment.* By John Hey Robertson, m.d.—*Glasgow*, 1842. 8vo, pp. 119.

Sometime since we noticed a previous work of Dr. Robertson, along with several other treatises on spinal affections. We then called attention to the efficacy of dry cupping in some of these cases, and approved of the mode of practising it which Dr. Robertson describes, (Br. & For. Med. Rev. No. XXIII. p. 183.) In the work before us, which the author informs us is a continuation of the former, the same practice is advocated, and a variety of cases illustrative of its efficacy are adduced. None of these which we have examined present any points of such novelty as to induce us to quote them at length. We are disposed to believe that many cases of rheumatism and the majority of cases of paralysis will be but very partially relieved by *dry* cupping alone; and that not a few of the former would be only aggravated by that treatment. Certain cases compounded of neuralgia and rheumatism, and cases of muscular rheumatism, may be relieved by this practice; but we apprehend that in cases of acute articular rheumatism, dry cupping would be worse than useless. In short, *dry* cupping is contra-indicated, wherever inflammatory action has prevailed, and where congestion, not simply passive but active, and accompanied with positive dilatation of the capillaries, has taken place. In such cases, if cupping-glasses are at all to be used, the armed ones must be selected. We observe, indeed, that in some cases, Dr. Robertson allows a few drops of blood to be drawn by the cups, after having first applied these in the dry mode.

Dr. Robertson has ventured, in the present volume, on the subject of "the theory of the remedial action of dry cupping," a task, which, considering how he has executed it, we think he might have declined with advantage to his own reputation. "The proper time," we are told (p. 89), "to keep a glass on is from 15 to 45 seconds. . . . . . . . If permitted to remain on for 10 or 15 minutes, considerable pain and even swelling will be the result."

Without retracting our former partial approval* of Dr. Robertson's earlier treatise, we must express our conviction that the apparent success of the author in many alleged cases of rheumatism and paralysis, detailed in the present volume, must be accounted for from errors of diagnosis.

In a literary point of view, the work is extremely defective, and has too much the air of having been designed for the general rather than for the professional reader. As a volume of medical reports, it is also very deficient. Thus in case 24, we are told that along with cupping, "internal medicine was prescribed!" What are we to understand by a phrase so unbusiness-like as this? May we not legitimately conjecture that this anonymous "internal medicine" may have had as great a share in the cure as the dry cupping, or perhaps a greater? Dr. Robertson, at least, has no right to call us uncharitable for forming such a conjecture.

* We cannot refrain from here noticing a species of dishonesty which certain publishers practise and authors permit; that, namely, of making garbled extracts from notices in reviews. In our remarks on Mr. R.'s former work, we say—"it is not a bad book, but it is not needed:" of which observation Mr. R. or his publisher quotes the first clause and leaves out the last, and continues the quotation as if nothing had been omitted. This is indefensible, and it is not the only misquotation.

